# Optimisation of a primary human CAR‐NK cell manufacturing pipeline

**DOI:** 10.1002/cti2.1507

**Published:** 2024-05-02

**Authors:** Aline Pfefferle, Julian Contet, Kahlia Wong, Charlotte Chen, Els Verhoeyen, Chloe K Slichter, Kimberly S Schluns, Joseph Cursons, Richard Berry, Iva Nikolic, Jai Rautela, Nicholas D Huntington

**Affiliations:** ^1^ Biomedicine Discovery Institute and the Department of Biochemistry and Molecular Biology Monash University Clayton VIC Australia; ^2^ oNKo‐Innate Pty Ltd Moonee Ponds VIC Australia; ^3^ CIRI, Université de Lyon, INSERM U1111, ENS de Lyon Université Lyon 1, CNRS, UMR 5308 Lyon France; ^4^ INSERM, C3M Université Côte d'Azur Nice France; ^5^ Kite Pharma, a Gilead Company Santa Monica CA USA

**Keywords:** immunotherapy, lymphocytes, NK cells, translational immunology

## Abstract

**Objectives:**

Autologous chimeric antigen receptor (CAR) T‐cell therapy of B‐cell malignancies achieves long‐term disease remission in a high fraction of patients and has triggered intense research into translating this successful approach into additional cancer types. However, the complex logistics involved in autologous CAR‐T manufacturing, the compromised fitness of patient‐derived T cells, the high rates of serious toxicities and the overall cost involved with product manufacturing and hospitalisation have driven innovation to overcome such hurdles. One alternative approach is the use of allogeneic natural killer (NK) cells as a source for CAR‐NK cell therapy. However, this source has traditionally faced numerous manufacturing challenges.

**Methods:**

To address this, we have developed an optimised expansion and transduction protocol for primary human NK cells primed for manufacturing scaling and clinical evaluation. We have performed an in‐depth comparison of primary human NK cell sources as a starting material by characterising their phenotype, functionality, expansion potential and transduction efficiency at crucial timepoints of our CAR‐NK manufacturing pipeline.

**Results:**

We identified adult peripheral blood‐derived NK cells to be the superior source for generating a CAR‐NK cell product because of a higher maximum yield of CAR‐expressing NK cells combined with potent natural, as well as CAR‐mediated anti‐tumor effector functions.

**Conclusions:**

Our optimised manufacturing pipeline dramatically improves lentiviral transduction efficiency of primary human NK cells. We conclude that the exponential expansion pre‐ and post‐transduction and high on‐target cytotoxicity make peripheral blood‐derived NK cells a feasible and attractive CAR‐NK cell product for clinical utility.

## Graphical abstract

Here we report an optimised chimeric antigen (CAR) natural killer (NK) cell manufacturing pipeline with class‐leading lentiviral transduction efficiency that yields clinically relevant doses of CAR‐NK cells. We have performed an in‐depth comparison of different primary human NK cell sources as a starting material for a clinical CAR‐NK cell product. Adult peripheral blood‐derived NK cells were identified to be the superior source for generating a CAR‐NK product because of high yield and potent natural, as well as CAR‐mediated anti‐tumor effector functions.

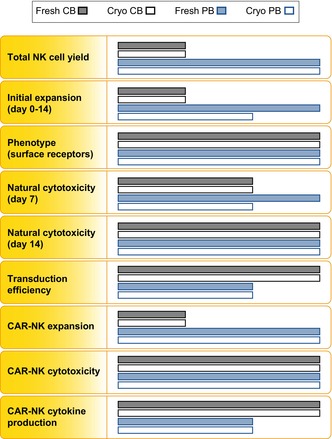

## Introduction

The clinical success of chimeric antigen receptor (CAR) T‐cell therapy against B‐cell malignancies has transformed the treatment landscape for such diseases. However, along with the deep and durable responses of CAR‐T‐cell therapy comes a high rate of serious side effects related to CAR‐T‐cell expansion and activation. Serious and sometimes fatal side effects are observed with both autologous and allogeneic CAR‐T‐cell therapy, such as neurotoxicity, immune effector cell‐associated neurologic syndrome (ICANS) and cytokine release syndrome (CRS). Of the patients receiving CAR‐T‐cell therapies, 37–93% develop CRS associated with elevated levels of IL‐6, IFNγ, GM‐CSF and soluble IL‐6R and IL‐2Rα.^1^, ^2^, ^3^, ^4^, ^5^ Subsequent development of ICANS occurs in 23–67% of patients with symptoms ranging from encephalopathy to expressive aphasia and seizures.^1^, ^3^, ^5^, ^6^, ^7^, ^8^, ^9^ Allogeneic CAR‐T‐cell therapy carries the additional risk of inducing graft versus host disease (GvHD),^10^, ^11^ resulting in > 75% of CAR‐T‐cell trials utilising autologous T cells. Genetically engineering allogeneic CAR‐T cells by knocking out the endogenous T‐cell receptor (TCR) is one strategy currently being investigated, but like using autologous T cells, this approach only further complicates the manufacturing process and prolongs time to treatment^12^ (clinicaltrials.gov).

These toxicity issues and manufacturing challenges associated with CAR‐T‐cell products have seen a pivot in early‐stage CAR‐cell therapy towards a safer allogeneic and potentially off‐the‐shelf product, namely, natural killer (NK) cells. NK cells are innate cytotoxic lymphocytes of relatively short lifespan compared to T cells and capable of spontaneously recognising and lysing malignant cells via an array of germline encoded receptors.^13^ The degree of NK cell infiltration in several tumor types is prognostic of patient outcome.^14^, ^15^, ^16^ Haematopoietic stem cell transplantation (HSCT) for acute myeloid leukaemia (AML) has highlighted the role of NK cells as potent contributor to the graft versus leukaemia (GvL) response.^17^ Furthermore, adoptive cell therapy (ACT) of allogeneic haplo‐identical NK cells has been clinically proven to be safe without the risk of inducing GvHD.^18^, ^19^ Indeed, the superior safety profile of NK cells versus T cells, was evident in the first anti‐CD19 CAR‐NK cell phase I trial utilising allogeneic cord blood‐derived NK cells with no serious toxicities observed, even in HLA‐unrelated recipients.^20^

Early CAR‐NK cell studies and clinical trials utilised NK‐92 cells, a NK lymphoma cell line, largely owing to the challenges of transducing and expanding primary human NK cells.^21^, ^22^, ^23^ Similarly, induced pluripotent stem cell (iPSC)‐derived NK (iNK) cells pose an appealing starting material for a CAR‐NK cell product because of the ease of manufacturing and off‐the‐shelf potential.^24^, ^25^, ^26^ NK‐92 and iNK cells have numerous phenotypic abnormalities compared to healthy adult NK cells including the lack CD16 expression and antibody‐dependent cellular cytotoxicity (ADCC), limiting their cytotoxic potential.^21^, ^27^, ^28^ To overcome this limitation, iNKs cells have been genetically engineered to express a non‐cleavable form of CD16, despite CD16 shedding playing an important role in serial killing.^29^, ^30^ *In vivo* persistence is also severely impacted in both cell types, with NK‐92 cells requiring irradiation prior to infusion.^21^, ^31^, ^32^

Primary allogeneic NK cells for adoptive cell transfer can be sourced either from umbilical cord blood (CB) or adult peripheral blood (PB). Supply of fresh cord blood is typically unpredictable, but cryopreserved CB units are readily available because of routine biobanking. Comparatively, adult PB‐derived NK cells are commonly isolated from buffy coats, a cell‐enriched by‐product of blood donations, and thus a readily accessible fresh source. Lastly, large‐scale PB‐derived NK cells can be obtained from healthy donor apheresis products, with a typical yield of ~3.5–7 × 10^8^ NK cells.^33^, ^34^

Adoptive NK cell therapy relies on *in vitro* expansion protocols to achieve sufficient cell numbers for *in vivo* treatment. While CAR‐T cells undergo clonal expansion upon antigen stimulation, leading to *in vivo* proliferation and persistence, this may not hold true for CAR‐NK cells^35^ because of divergent biology between cell types. While this characteristic is an important contributor to the increased safety profile of CAR‐NK cells, it also highlights the need for a high degree of *in vitro* cellular expansion prior to treatment. The inverse relationship between NK cell maturation and proliferative capacity in response to cytokine stimulation^36^ posits that CB‐derived NK cell would have a higher proliferative capacity than PB‐derived NK cells when expanded in cytokine alone. However, cytokine‐induced *in vitro* expansion of primary NK cells is limited and insufficient for a clinical NK cell adoptive therapy product.^37^ Fortunately, feeder‐based expansion protocols have been developed to significantly increase primary NK cell *in vitro* expansion and also improve NK cell viability and fitness at the end of the process.^38^, ^39^, ^40^

There have been several challenges faced when using adult PB‐derived NK cells for CAR‐NK cell manufacturing, thus preclinical and clinical studies to date have largely relied on NK‐92 cells, CB‐derived NK cells or iPSC‐derived NK cells. Based on their superior function and accessibility, we hypothesized that PB‐derived NK cells would present the ideal starting material for a potent CAR‐NK cell product if these manufacturing limitations could be overcome. To this end, we have developed an optimised expansion and transduction protocol for primary human NK cells which can easily be scaled‐up for commercial use. We have performed a detailed comparison of both fresh and cryopreserved cord‐ and adult peripheral blood‐derived CAR‐NK cells, characterising their phenotype, functionality, expansion potential and transduction efficiency at crucial timepoints of our CAR‐NK manufacturing pipeline. NK cell yield per unit of blood was significantly increased in adult peripheral blood compared to cord blood and PB‐derived NK cells also exhibited superior recovery after short‐term cryopreservation. No phenotypic or functional differences were observed between fresh and short‐term cryopreserved NK cells irrespective of source, but long‐term cryopreservation did impact cellular fitness, leading to overall reduced proliferative capacity and reduced natural cytotoxicity on day 7. Efficient lentiviral transduction combined with exponential expansion pre‐ and post‐transduction and high on‐target cytotoxicity makes fresh and cryopreserved PB‐derived NK cells a feasible and attractive CAR‐NK cell product for future clinical testing.

## Results

### Superior yield and recovery after cryopreservation of PB‐NK cells over CB‐NK cells

**Figure 1:**
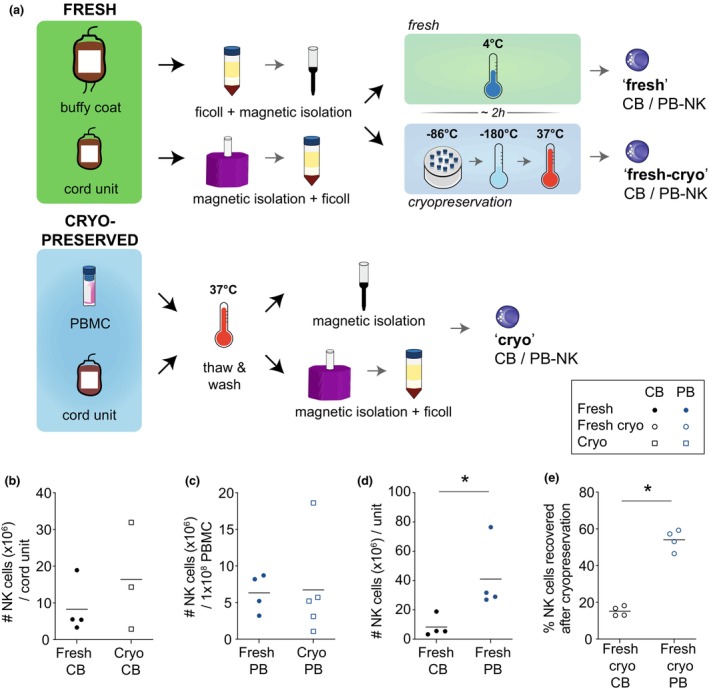
NK cell yield from different blood sources. Overview of the NK cell isolation protocol from fresh and cryopreserved blood sources **(a)**. Total NK cell yield from fresh or cryopreserved cord blood units **(b)**. Yield of NK cells isolated from 1 × 10^8^ PBMCs purified from fresh buffy coats or from frozen PBMCs **(c)**. Yield of NK cells isolated from fresh buffy coats or fresh cord blood **(d)**. Percentage of NK cells recovered after short‐term cryopreservation of NK cells isolated from fresh buffy coats or fresh cord blood. % recovery = (# of NK cells after thawing/# of NK cells cryopreserved) * 100 **(e)**. *n* = 3–5. Statistical analysis was performed using Prism 8 (GraphPad Software Inc, San Diego). Significance was calculated using either a Mann–Whitney *U*‐test or a Wilcoxon signed rank test, with multiple *t*‐tests (Benjamini) being used when doing multiple two‐group comparisons within the same dataset. Linear regression analysis was performed followed by a Spearman *r* test to determine significance. *P*‐values: * < 0.05. Error bars represent standard deviation.

To accurately and fairly compare cord blood to adult peripheral blood as an NK cell starting source, we tested fresh (‘fresh’), short‐termed cryopreserved (‘fresh‐cryo’) and long‐term cryopreserved (‘cryo’) NK cells isolated from each source (Figure 1a). Long‐term cryopreserved peripheral blood mononuclear cells (PBMCs) and cord units had been stored in liquid nitrogen anywhere from a few months to 5 years prior to isolation. Fresh NK cells were isolated within 24–48 h after product formulation because of logistical issues in terms of supply and experimental setup. Owing to high donor variability, particularly in terms of proliferative potential, freshly isolated NK cells were short‐term cryopreserved after isolation. This allowed for a side‐by‐side comparison of fresh and cryopreserved NK cells from the same donor, eliminating any confounding donor differences.

The NK cell content of umbilical cord blood units varied greatly, ranging from 3.3–18.9 × 10^6^ cells per fresh cord unit to 2.9–31.9 × 10^6^ cells per cryopreserved cord unit (Figure 1b). Similarly, a range in PBMC number and NK cell frequency was observed in fresh buffy coats (~1 × 10^9^ PBMCs), with the NK cell yield ranging from 3.2–8.7 × 10^6^ cells to 3.1–18.6 × 10^6^ cells isolated from either 10^8^ fresh or cryopreserved PBMCs, respectively (Figure 1c). Fresh buffy coats contained significantly more NK cells than fresh cord units (Figure 1d). Cryopreservation of freshly isolated NK cells was well tolerated when PB‐derived, with ~50–60% of cells recovered after cryopreservation while only ~15% of CB‐derived NK cells could be consistently recovered (Figure 1e).

Natural killer cell yield, while highly variable between donors, was comparable between fresh and cryopreserved starting source material. Cryopreservation of freshly isolated NK cells was well tolerated by PB‐derived NK cells; however, recovery was severely reduced in CB‐derived NK cells. As such, total NK cell yield per unit of blood, was ~6× greater for PB than for CB, which can be further increased 30–70× for an adult apheresis product.

### Characterisation of a CB‐ and PB‐derived feeder‐expanded NK cell product

To achieve clinically relevant *in vitro* expansion, we optimised a feeder‐based expansion protocol for primary NK cells. Stimulation with irradiated K562 cells in the presence of IL‐15 and IL‐21, every 7 days over a 28‐day period significantly increased the fold expansion of PB‐derived NK cells compared to NK cells expanded in only IL‐15 (~600× to 4×) (Supplementary figure 1a). Using the genetically modified K562 cell line (CSTX002) as feeder cells further increased the total fold expansion compared to parental K562 cells (Supplementary figure 1b). Optimisation of the NK to feeder cell seeding ratio yielded increased fold expansion when seeded at a 1:2 ratio, particularly in the second week of expansion (Supplementary figure 1c). At a 1:2 NK to feeder ratio, CB‐derived NK cells had poorer expansion over the initial 7 days than the PB‐derived NK cells, as well as lower expansion during the second week (Supplementary figure 1d). CSTX002 is a K562 clone that has been modified to express CD64, CD86, 41BBL, truncated CD19 and membrane‐bound IL‐21 (mbIL‐21) and induces robust NK cell expansion largely because of its increased expression of 41BBL and the presence of IL‐21.^41^, ^42^ Based on these findings, we generated a simplified NK‐specific feeder cell lines, by overexpression 41BBL and mbIL‐21 on K562 cells. We observed a clear positive correlation between the level of 41BBL expression of different feeder clones and the NK cell expansion observed after 7 days of co‐culture (Supplementary figure 1e). Clone KE2‐F9 was found to express similarly high levels of 41BBL as CSTX002 cells and resulted in similar, class‐leading fold expansion to CSTX002 of either fresh or cryopreserved PB‐derived NK cells (Supplementary figure 1f). As such, all further experiments were performed using NK cells expanded on KE2‐F9 feeder cells.

**Figure 2:**
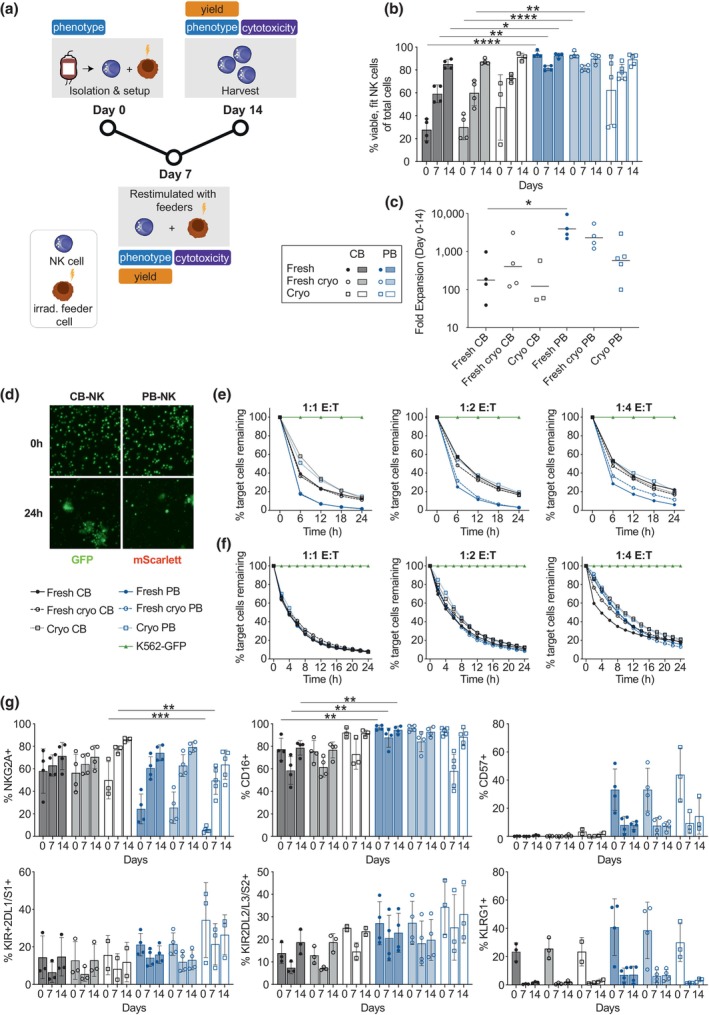
Proliferative, functional, and phenotypic characterisation of NK cells from different blood sources. Overview of experimental timeline and methodology **(a)**. Percentage of viable, fit NK cells (CD56^+^Granzyme B^+^) out of total cells analysed by flow cytometry at days 0, 7 and 14 of culture for NK cells isolated from varying sources **(b)**. Total fold expansion over 14 days of culture of NK cells isolated from varying sources **(c)**. Representative IncuCyte images of fresh CB‐ and PB‐derived NK cells at timepoint 0 and after 24 h of co‐culture with target cells (K562‐GFP) at a 1:1 E:T ratio **(d)**. Percentage of target cells (K562‐GFP), enumerated by object count and normalised to target cells only (K562‐GFP), remaining over 24 h of co‐culture with day 7 **(e)** or day 14 **(f)** expanded NK cells from varying sources at three E:T ratios (1:1, 1:2, 1:4). Characterisation of surface receptor expression in NK cells expanded over 14 days from varying sources **(g)**. *n* = 2–5. Statistical analysis was performed using Prism 8 (GraphPad Software Inc, San Diego). Significance was calculated using either a Mann–Whitney *U*‐test or a Wilcoxon signed rank test, with multiple *t*‐tests (Benjamini) being used when doing multiple two‐group comparisons within the same dataset. Linear regression analysis was performed followed by a Spearman *r* test to determine significance. *P*‐values: * < 0.05, ** < 0.01, *** < 0.001, **** < 0.0001. Error bars represent standard deviation.

After NK cell isolation (Figure 1a), the cells were seeded with irradiated KE2‐F9 feeders at either a 1:1 (CB) or 1:2 (PB) NK to feeder ratio, a process that was repeated on day 7. On day 7, prior to restimulation, and on day 14, the cells were counted and characterised in terms of phenotype and functionality (Figure 2a). Fresh CB‐derived NK cells and fresh‐cryo CB‐derived NK cells had significantly decreased viability and cellular fitness after isolation compared to fresh and fresh‐cryo PB‐derived NK cells, respectively. NK cell fitness was defined by high levels of CD56 and Granzyme B amongst viable NK cells (Supplementary figure 2). Cellular fitness slowly increased by day 7, almost reaching PB‐derived fitness by day 14 (Figure 2b). Notably, short‐term cryopreservation had no effect on cellular fitness in CB nor PB‐derived NK cells. In line with lower cellular fitness, significantly lower expansion was observed in fresh CB‐derived NK cells than in fresh PB‐derived NK cells (Figure 2c). Similar trends were observed for fresh‐cryo and cryo CB‐ compared to PB‐derived NK cells, with PB‐derived NK cells exhibiting superior expansion potential to that of CB‐derived NK cells over a 14‐day culture period (Figure 2c). Natural cytotoxicity was significantly increased in fresh and fresh‐cryo PB‐derived NK cells compared to cryo PB‐derived NK cells at different E:T ratios on day 7 (Figure 2d and e). A trend for increased cytotoxic capacity was also observed when comparing fresh/fresh‐cryo PB‐derived NK cells to CB‐derived NK cells on day 7 (Figure 2d and e). These differences in natural cytotoxicity were abrogated by day 14 of culture after the two rounds of feeder stimulation (Figure 2f). Phenotypic differences (largely reflecting differences in maturation state) between CB‐ and PB‐derived NK cells were diminished following 14 days of culture, with PB‐NK cells upregulating NKG2A and downregulating CD57 and KLRG1 expression over time, while the fraction of CD16 and KIR expressing NK cells was stable (Figure 2g). Short‐term cryopreservation did not impact surface receptor expression in CB‐ nor PB‐derived NK cells compared to fresh counterparts (Figure 2g).

### Efficient transduction of primary NK cells with an optimised CAR vector

Primary NK cells are innately resistant to viral infection, making them a difficult entity to transduce efficiently. Despite significant upregulation of LDLR expression on day 7 cultured NK cells (Supplementary figure 3a), transduction with a VSV‐G pseudotyped lentivirus only resulted in low levels of transduction even at high multiplicity of infection (MOI) with a number of different promoter regions tested (Supplementary figure 3b, c). In line with previous studies,^43^, ^44^ transduction efficiency with baboon‐enveloped (BaEV) pseudotyped lentivirus significantly increased transduction efficiency even at low MOI (Supplementary figure 3d, e). Using the MSCV promoter consistently resulted in the highest level of transduction efficiency (% mScarlett^+^CD19 CAR^+^), achieving ~60% transduction at a MOI of 5 (Supplementary figure 3d–g).

**Figure 3:**
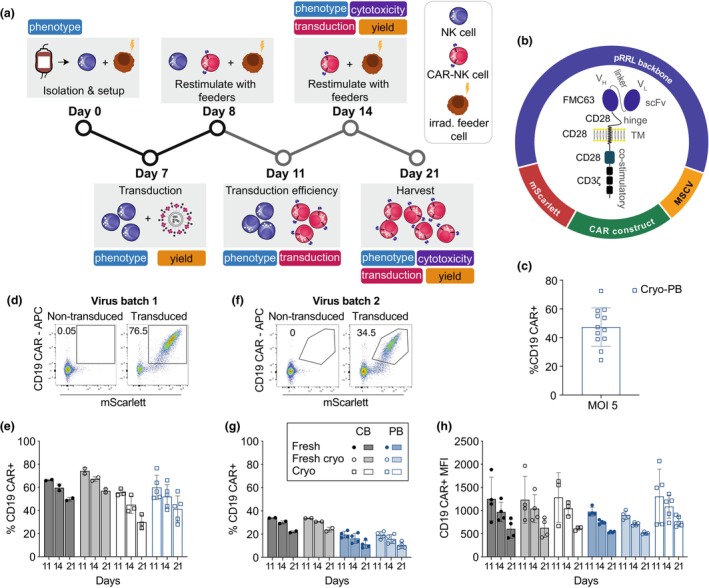
Transduction efficiency of NK cells from varying sources. Overview of experimental timeline and methodology **(a)**. Schematic of viral plasmid used for transduction and architecture of the CAR construct **(b)**. Transduction efficiency of CB‐derived NK cells **(c)**. Representative flow cytometry plots of non‐transduced and transduced NK cells on day 11 after isolation using virus batch 1 **(d)** and batch 2 **(f)**. Percentage of CAR+ NK cells (CD19 CAR^+^mScarlett^+^) out of total NK cells over time (days 11–21) transduced with virus batch 1 **(e)** and batch 2 **(g)**. Mean fluorescent intensity of CD19 CAR expression by NK cells over time (days 11–21) **(h)**. *n* = 2–36.

By combining our optimised protocols for transduction and expansion, we developed an optimised pipeline for large‐scale CAR‐NK cell production using primary NK cells isolated from fresh or cryopreserved cord or adult peripheral blood (Figure 3a). Day 7 feeder‐expanded NK cells were transduced with an anti‐CD19 CAR construct (Figure 3b), followed by restimulation with fresh feeder cells on days 8 and 14 of culture (Figure 3a). Phenotypic and functional characterisation of the CAR‐NK cell product was performed on days 14 and 21 of culture (Figure 3a). Transduction efficiency of primary NK cells was viral batch‐dependent, ranging from 15 to 77%, with an average of 45% (Figure 3c). Short‐term cryopreservation did not affect transduction efficiency of CB or PB‐derived NK cells, and very little donor variation was observed (Figure 3e and g). On average, a 10% increase in transduction efficiency was observed in CB‐ over PB‐NK cells, but this was not significant (Figure 3e and g). However, we did observe differences in transduction efficiency as a result of viral batch variation (~70% to ~30%), most likely because of the virus being titrated on HEK 293 T cells instead of a lymphoid cell lineage (Figure 3d–g). As a result of the logistical challenge of obtaining fresh cord units and buffy coats on the same day, all fresh and fresh‐cryo PB‐NK cells were transduced with the less efficient viral batch (Figure 3e–g). Further expansion of the CAR‐NK cells resulted in a low but steady decrease in the frequency of CAR+ NK cells in culture, in line with a reduction in surface density (MFI) (Figure 3e, g and h).

Optimisation of promoter and viral envelope identified an efficient lentiviral construct leading to high and sustained transduction efficiency of both fresh and cryopreserved CB‐ and PB‐derived NK cells with minimal donor variation.

### Sustained proliferation and no phenotypic perturbation in CAR‐transduced NK cells

**Figure 4:**
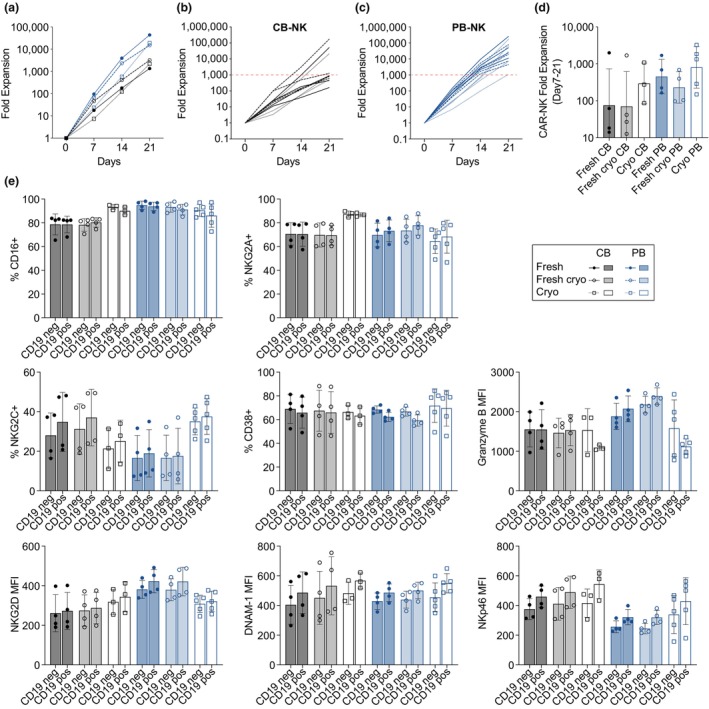
Proliferative potential and phenotype of CAR‐NK cells from varying sources. Total fold expansion of CAR‐NK cells over 21 days, summarised by NK cell source in **(a)**, or separated into individual donors based on starting source, CB‐derived **(b)** or PB‐derived **(c)**. Total fold expansion of CAR‐NK cells from varying sources after transduction (days 7–21) **(d)**. Characterisation of surface receptor expression and intracellular Granzyme B levels of transduced NK cells on day 14 of culture, stratified based on CAR expression (CAR neg/pos) **(e)**. *n* = 3–5.

Transduction of primary NK cells with an anti‐CD19 CAR did not impact expansion potential in response to repeated feeder stimulation over the subsequent 14 days examined (Figure 4a). With the exception of two CB donors, CB‐derived NK cells expanded < 1000‐fold over 21 days, while PB‐derived NK cells, on average, expanded 10 000 to 100 000‐fold in the same time frame (Figure 4b and c). The similar trend was observed with CAR‐NK‐specific expansion, measured from the point of transduction (day 7) onwards.

Phenotypic characterisation on days 11, 14 and 21 showed no adverse effects on CAR‐expressing NK cells, regardless of source (Supplementary figure 4a). We observed no trends in transduction efficiency and baseline NK cell surface receptor expression nor metabolic activity, as we observed no difference between non‐transduced (NT) and transduced (MOI 5) NK cells on day 11 across all NK cell sources examined (Supplementary figure 4b). Similarly, we observed no differences between CAR‐expressing (CD19 CAR pos) and CAR negative (CD19 CAR neg) cells within the same donor on day 14 (Figure 4e) and day 21 (Supplementary figure 4c). The absence of any inhibitory (NKG2A) and activating receptor (DNAM‐1, CD16, NKG2C, NGK2D, NKp46) changes as well as no impact on metabolic receptors (CD38) and effector molecules (Granzyme B) led us to conclude that no tonic signalling via the CAR was occurring. This was further confirmed by the absence of spontaneous degranulation in CAR‐NK cells (data not shown).

Chimeric antigen receptor‐transduced primary NK cells are not phenotypically altered and maintain their proliferative capacity leading to efficient expansion of a CAR‐NK cell population.

### Elimination of antigen‐specific target cells by CAR‐NK cells without IL‐6 production

**Figure 5:**
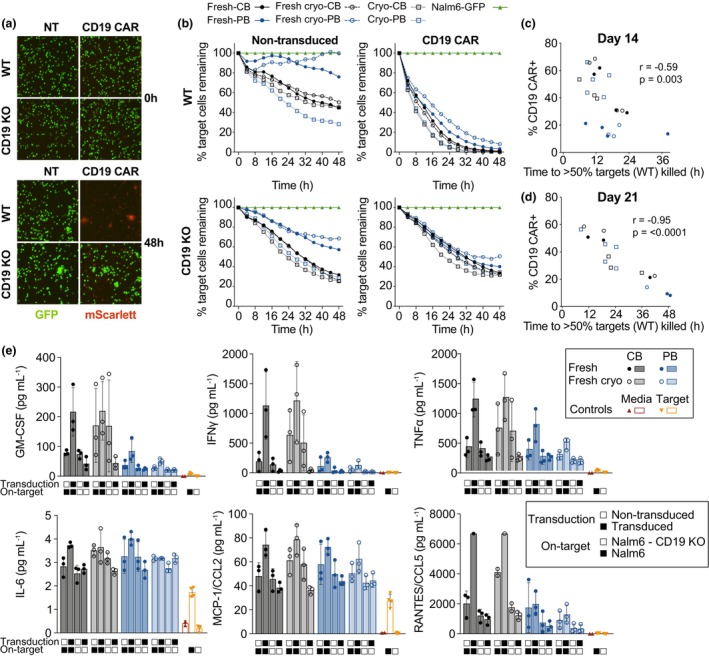
Cytotoxic capacity of CAR‐NK cells from varying sources. Representative IncuCyte images of non‐transduced (NT) and transduced (CD19 CAR) NK cells at timepoint 0 and after 48 h of co‐culture with WT or CD19 KO (Nalm6‐GFP) target cells at a 1:2 E:T ratio. CD19 CAR‐expressing NK cells are mScarlett^+^ **(a)**. Percentage of target cells (WT or CD19KO Nalm6‐GFP), enumerated by object count and normalised to target cells only (NALM6‐GFP), remaining over 48 h of co‐culture with day 14 non‐transduced (NT) or transduced (CD19 CAR) NK cells from varying sources at a 1:2 E:T ratio **(b)**. Relationship between the time required to kill > 50% of Nalm6‐GFP target cells and the frequency of CAR+ NK cells in the effector population at day 14 **(c)** and 21 **(d)** of culture at an E:T ratio of 1:2. Quantification of cytokines and chemokines produced during a 48 h cytotoxicity assay by day 14 expanded transduced and non‐transduced NK cells against WT and CD19 KO Nalm6 target cells, stratified based on NK cell source **(e)**. For on‐target CB‐transduced NK cells, the maximum quantifiable value for RANTES/CCL5 is plotted, as values exceeded a standard curve. *n* = 2–23.

Wild‐type (WT) Nalm6 cells, a B‐cell precursor leukaemia cell line expressing CD19, and a CD19 KO Nalm6 clone were used to test on‐target cytotoxicity of our CAR‐NK cell products after 14 and 21 days of culture (Figure 5a). Non‐transduced day 14‐expanded NK cells were only able to, at best, control the growth of WT and CD19 KO‐Nalm6, while CAR‐NK cells efficiently eliminated all WT, but not CD19 KO target cells within 48 h (Figure 5a and b). The rate of target cell elimination correlated with the frequency of CAR+ NK cells within each sample at both day 14 (Figure 5c) and 21 (Figure 5d). Although day 14 fresh and fresh‐cryo PB‐derived NK cells had a low frequency of CAR+ NK cells, their on‐target cytotoxicity was similar to CB‐derived CAR‐NK samples containing 2–3× as many CAR+ NK cells (Figure 5c). A higher frequency of CAR+ NK cells also resulted in successful target elimination at a 1:4 E:T ratio within 48 h (Supplementary figure 5a) but was not fully achieved in samples with lower transduction efficiency (Supplementary figure 5b). On‐target cytotoxicity was maintained on day 21, but the rate of target cell elimination was consistently dependent on the frequency of CAR‐NK cells within the sample (Figure 5d, Supplementary figure 5c and d). To examine the durability of our CAR‐NK cell product, cryo PB‐derived NK cells were exposed to repeated target cell additions every 24 h for a total of 7 additions. While the rate of on‐target cytotoxicity slowly diminished over repeated additions, it was only after the 7th serial challenge that the CAR‐NK cells became exhausted and unable to eliminate Nalm6 cells within the 24 h time frame (Supplementary figure 5e).

Supernatants from the cytotoxicity assays were collected after 48 h and quantified for chemokines and cytokines to identify any potential systemic effects our CAR‐NK cell product may incur *in vivo*. In line with the on‐target cytotoxicity, we observed a trend for increased production of IFNγ and TNFα in CAR‐NK/WT‐Nalm6 wells (Figure 5e). The apparent increased production of particularly IFNγ, but also CCL5, in CB‐derived samples can be attributed to the increased frequency of CAR‐NK cells present in those cultures compared to the PB‐derived samples (Figure 5e). Additionally, we observed a general production of GM‐CSF, CCL2 and IL‐2 by day 14‐expanded NK cells regardless of CAR expression (Figure 5e, Supplementary figure 5f). Importantly, only background levels of IL‐6 were detected in all conditions (Figure 5e).

Surface expression of the anti‐CD19 CAR translated to efficient on‐target cytotoxicity with increased IFNγ, TNFα, CCL5 and no IL‐6 production. The rate of on‐target cytotoxicity correlated with frequency of CAR expression and was maintained even after 6 repeated target cell challenges. A general increase in GM‐CSF, CCL2 and IL‐2 was detected in our feeder‐expanded NK cells, highlighting a favorable cytokine/chemokine profile for *in vivo* treatment.

## Discussion

Our in‐depth comparison of fresh and cryopreserved CB‐ and PB‐derived CAR‐NK cells using our advanced platform has identified adult PB‐derived NK cells as a highly attractive cell source for a CAR‐NK cell product. Our optimisation of the lentiviral construct and *in vitro* expansion protocol have eliminated the major limitations of manufacturing an allogeneic PB‐derived CAR‐NK cell product for clinical use. In contrast, sensitivity to cryopreservation, restricted starting cell number and reduced overall expansion of CB‐NK cells are clear limitations for the manufacturing of CB‐derived CAR‐NK cells using our protocol.

Natural killer cells are inherently resistant to viral infection. As a result, viral transduction of primary NK cells is often highly inefficient, representing a major limitation in generating a stable CAR‐NK cell product. In line with previous reports,^43^, ^44^ we observed a substantial increase in transduction efficiency with Baboon envelope (BaEV) compared to the conventional vesicular stomtatitis virus type‐G (VSV‐G) pseudotyped lentivirus, even at much lower MOIs. Transduction was performed on day 7 of culture, as activation is required for expression of the viral receptors LDLR (VSV‐G) and ASCT‐2 (BaEV) on NK cells.^44^, ^45^, ^46^ Additionally, day 7 of culture represented a timepoint whereby all feeder cells were eliminated from culture and NK cells were in their peak proliferative phase. Importantly, lentiviral transduction on day 7 did not impede NK cell proliferation in response to fresh feeder stimulation on day 8 of culture.

To further boost transduction efficiency, we optimised the lentiviral construct by testing a variety of different promoter sequences, which has previously been shown to affect transduction efficiency for VSV‐G pseudotyped lentivirus.^47^ It is speculated that transcriptional silencing of the promoter can contribute to the generally low transduction efficiency observed in primary NK cells.^47^ Intriguingly, we observed no anti‐CD19 CAR surface expression under the CMV promoter, despite average levels of lentiviral integration as observed by mScarlett expression. Highest transduction efficiency was observed under the MSCV promoter, which achieved on average 45% transduction in primary NK cells at a MOI of 5, but could be as high as 77%. Very little donor variation was observed in terms of transduction efficiency, in line with all NK cell subsets transducing equally well. Phenotypically, no difference was observed between transduced and non‐transduced cells, nor CAR negative and positive cells within the same donor. Instead, the biggest variation in transduction efficiency was related to virus preparation and viral titre determination. Titration of individual viral batches was performed on HEK 239 T cells which did not consistently translate to primary NK cells. Performing viral titrations on lymphocyte cell lines is likely to result in a more accurate titration and thus more consistent transduction levels.

Contrary to previous reports, NK cell viability and subsequent proliferation was not impacted by transduction on day 7 of culture,^48^ most likely attributed to our robust feeder‐based expansion protocol. We generated our own NK‐specific feeder cell line based on previous reports highlighting the synergy between 41BBL and membrane‐bound IL‐21 expression.^41^ A clear correlation between the level of 41BBL expression and NK cell expansion was observed, achieving comparable expansion levels as the commonly used CSTX002 feeder cell line which also overexpresses a number of additional, but not NK cell relevant surface proteins. Restimulation with feeder cells directly after overnight transduction retained both transduced and non‐transduced cells on the proliferative trajectory observed during the initial 7 days of culture. This culminated in 75–295‐fold expansion for CB‐derived CAR‐NK cells and 229–806‐fold expansion for PB‐derived CAR‐NK cells between day 7 and 21 of culture.

We did note a small, but steady decrease in the intensity of CAR expression and the frequency of CAR‐expressing NK cells over the expansion period which was reflected in the degree of on‐target cytotoxicity. The cause of this loss in CAR expression over time is currently unknown. Inhibitory and activating receptor expression, as well as metabolic markers and granzyme B expression was identical between CAR negative and CAR positive NK cells and we observed no signs of tonic signalling because of CAR receptor aggregation. Nonetheless, in a serial challenge assay whereby our anti‐CD19 CAR‐NK cell product was repeated challenged with fresh CD19^+^ and CD19^−^ target cells every 24 h, we only observed a significant drop in on‐target cytotoxicity after the 7th serial challenge whereas natural/off‐target cytotoxicity is lost after the 3rd serial challenge. Despite a slight trend towards increased transduction efficiency in CB‐ compared to PB‐derived NK cells, on a per cell basis, PB‐derived anti‐CD19 CAR‐NK cells were more potent at killing CD19^+^ Nalm6 cells after 14 days of culture.

While NK cells express a range of potent effector molecules, their ability to coordinate a robust anti‐tumor response is driven in part by the wide range of cytokines and chemokines which they secrete upon activation. The cytokine/chemokine profile of CAR‐NK cells after on‐target interaction is of clinical relevance because of the serious and potentially lethal side effects observed with CAR‐T‐cell therapy, namely, CRS and ICANS. The first in‐human CAR‐NK cell trial reported no serious side effects and no increased serum cytokine levels.^20^ Similarly, we observed no significant and meaningful change in the relative release of IL‐6 after target interaction. On the contrary, we observed increased release of GM‐CSF, IFNγ, TNFα, MCP‐1/CCL2 and RANTES/CCL5 after on‐target CAR‐NK cell interaction which mirror the level of CAR expression. Of note, IFNγ can play a role in inhibiting the growth of tumor cells; however, it also plays a role in promoting full activation of different immune populations as well as MHC‐I peptide expression required for subsequent T‐cell detection and killing.^49^, ^50^ Similarly, TNFα can promote cell death of target cancer cells, while a range of immune cells express TNFR2 and intracellular signalling molecules which can mediate NF‐κB activation and immune cell function.^51^ GM‐CSF is produced by activated NK cells and can promote inflammation mediated by myeloid cells as well as differentiation and function of dendritic cells.^52^, ^53^ Likewise, the chemokine RANTES (CCL5) is particularly important for the recruitment of CCR5‐expressing T‐cell populations to sites of inflammation.^54^ Our data demonstrate the ability of anti‐CD19 CAR to drive efficient activation of CAR‐NK cells in the presence of CD19‐expressing target cells such as the Nalm6 cell line, leading to a large increase in the expression of anti‐tumor cytokines as well was chemokines which are necessary for an effective immune recruitment cascade.

The increased safety profile of CAR‐NK cells over CAR‐T cells has high clinical relevance, as does the off‐the‐shelf potential. Adoptive cell therapy of haplo‐identical NK cells is both safe and effective, with KIR‐ligand mismatching further augmenting the GvL potency by reducing inhibitory signalling.^18^, ^19^, ^55^, ^56^ This drastically increases the potential donor pool from which a CAR‐NK cell product can be manufactured, instead of having to rely on a patient‐derived T cells with suboptimal fitness.^57^ Logistically, the supply of cord blood units is limited to cryopreserved biobanks, utilising standardised cord cryopreservation protocols, whereas adult peripheral blood can be readily sourced from healthy blood donors. Utilising a standardised cryopreservation protocol, we observed an increased sensitivity towards cryopreservation in CB‐derived NK cells compared to PB‐NK cells (‘fresh‐cryo’). Natural cytotoxicity was also significantly reduced in long‐term cryopreserved CB‐derived NK cells, while short‐term cryopreservation significantly impacted cell viability and total number compared to PB‐derived NK cells. As CB‐derived NK cells already exhibited lower overall expansion in a feeder‐based system, despite optimisation of the NK to feeder ratio, a further reduction in the source material as a result of cryopreservation will significantly impact the total yield. Our results are contrary to other studies which have observed increased proliferation in CB‐derived NK cells.^48^ This can largely be attributed to differences in expansion protocol, as CB‐derived NK cells are more responsive to cytokine stimulation. Cytokine stimulation alone, however, does not yield clinically relevant cell numbers and furthermore rapidly leads to cytokine dependence and cellular exhaustion, resulting in limited *in vivo* persistence. Feeder‐based expansion protocols circumvent these negative proliferation‐induced cellular phenotypes, most likely by a combination of diverse receptor input combined with lower, biologically relevant cytokine stimulation.

The first in‐human CAR‐NK cell trial utilised feeder‐expanded, and IL‐2 stimulated cryopreserved CB‐derived CAR‐NK cells. At its highest dose, 10^7^ cells kg^−1^ were infused.^20^ Assuming an average adult weight of 75 kg, our CAR‐NK manufacturing protocol can generate in the span of 14 days, with an average transduction efficiency of > 60%, 2 doses of cryopreserved CB‐derived CAR‐NK cells, 420 doses of PB‐derived (buffy coat) CAR‐NK cells or 2759 doses of fresh PB‐derived (apheresis) CAR‐NK cells. Factoring in cell loss because of cryopreservation, short‐term cryopreservation of apheresis‐isolated NK cells would still yield 809 doses with long‐term cryopreservation generating 204 doses of CAR‐NK cells. Overcoming the shortened *in vivo* persistence of adoptively transferred NK cells by giving repeated infusions of the product is thus highly feasible, without the need of utilising iPSC‐derived NK cells. iPSC‐derived NK cells are often described as an appealing starting material for a CAR‐NK product because of their ease of manufacturing and their unmatched proliferative capacity, which can overcome their poor *in vivo* persistence through repeated infusions.^24^, ^25^, ^26^ Although current cryopreservation protocols of expanded NK cells are limited, advancements in this field will solidify the off‐the‐shelf potential of PB‐derived CAR‐NK cells. In summary, we report an optimised CAR‐NK cell manufacturing pipeline with class‐leading lentiviral transduction efficiency that yields clinically relevant doses of adult PB‐derived CAR‐NK cells with potent on‐target serial killing.

## Methods

### Source material

#### Fresh source

Fresh cord blood was obtained by informed consent from the Murdoch Children's Research Institute and fresh buffy coats from healthy blood donors were obtained from the Australian Red Cross. Fresh cord blood was processed within ~48 h of production formulation and fresh buffy coats were processed within ~24 h of product formulation. Ethical approval was obtained from the Monash University Human Research Ethics Committee (Project ID: 25946).

#### Cryopreserved source

Peripheral blood mononuclear cells were isolated from fresh buffy coats by density gradient centrifugation (Ficoll‐Paque PLUS, Cytiva) and cryopreserved in 10% DMSO + 90% heat‐inactivated FCS (ThermoFisher), hereafter termed ‘cryo PB’. Cryopreserved cords, hereafter termed ‘cryo cord’ were cryopreserved at the Murdoch Children's Research Institute and obtained in a frozen state.

### Cell processing and culture

#### CB‐derived NK cells

Natural killer cells were isolated from either fresh or cryopreserved cord blood using the MACSxpress Whole Blood NK cell isolation kit (Miltenyi Biotec). Cryopreserved cord blood was quickly thawed in a 37°C water bath and diluted in PBS (Gibco, ThermoFisher) to a final volume of 400 mL. After centrifugation at 400 *g* for 10 min at room temperature (RT), the supernatant was removed, cells were pooled and washed in another 50 mL of PBS, centrifuged at 400 *g*/10 min/RT. The cell pellet was then resuspended in 30 mL of fresh PBS and NK cells were isolated using the same protocol as fresh cord blood. After negative magnetic isolation, the cell pellet was resuspended in 3 mL of RPMI‐1640 (Gibco, ThermoFisher) + 10% heat‐inactivated FCS and carefully layered onto 2 mL of Ficoll. The cells were centrifuged at 700 *g* for 20 min at RT (acceleration/deceleration at 3). The NK cell layer was collected and washed in 10 mL of PBS (400 *g*/5 min/RT) to yield the final purified cord‐derived NK cell starting material.

#### PB‐derived NK cells

Fresh buffy coats were diluted in PBS and carefully layered onto Ficoll for PBMC isolation by density gradient centrifugation (400 *g*/40 min/RT, acceleration/deceleration at 0). The PBMC layer was harvested into 2 × 50 mL tubes and washed in 50 mL PBS each, centrifuged at 400 *g*/10 min/RT. The cell pellet was resuspended in fresh 50 mL PBS and centrifuged at 300 *g*/5 min/RT. The cell pellets were then pooled and washed in a final 50 mL PBS at 200 *g*/10 min/RT to yield the isolated PBMCs.

Cryopreserved PBMCs were quickly thawed in a 37°C water bath and transferred into 10 mL of cold RPMI‐1640 + 10% heat‐inactivated FCS. Cells were centrifuged at 300 *g*/5 min/RT and washed in a further 10 mL of media prior to isolating NK cells.

NK cells were isolated from fresh or cryopreserved PBMCs using the NK cell isolation kit, human (Miltenyi Biotec) and magnetic LD columns (Miltenyi Biotec) to yield the final purified PB‐derived NK cell starting material.

#### ‘Fresh’ and ‘fresh‐cryo’ NK cells

Isolated NK cells from fresh cords or buffy coats were cryopreserved in cold CryoStor10 (Sigma) using a pre‐cooled Mr Frosty (−1°C/min, ThermoFisher). Once cells had reached −86°C, vials were dipped into liquid nitrogen for 5 s and then kept at −86°C for 10–20 min prior to thawing (as previously described for cryopreserved PBMCs), hereafter termed ‘fresh‐cryo’. ‘Fresh’ NK cells from the same donor were kept on ice for the duration of the cryopreservation process or alternatively freshly isolated from the remaining cord blood if the initial yield was too low.

#### Feeder/NK cell cultures

Unless specifically stated, NK cells were expanded using irradiated feeder cells as described below. Feeder cells, modified K562 cells (ATCC) expressing 41BBL, membrane‐bound IL‐21 and mScarlett (termed iKE2‐F9), were previously irradiated at 100 Gy, aliquoted and cryopreserved in 10% DMSO (Sigma) + 90% heat‐inactivated FCS. iKE2‐F9 cells were thawed and seeded at either 1:1 (cord‐derived NK) or 1:2 (PB‐derived NK) NK to feeder ratio, with a total of 0.5 × 10^6^ cells seeded in a 48‐well G‐Rex well (Wilson Wolf Manufacturing) in GMP‐SCGM (CellGenix) + 10% human serum (Sigma) + GlutaMax (Gibco, ThermoFisher) + 5 ng mL^−1^ IL‐15 (premium grade, Miltenyi Biotec). Half of the media was refreshed as needed. Cells were reseeded at initial seeding density on day 7 and 14 with fresh iKE2‐F9 feeders. Fold expansion was calculated by multiplying the weekly fold expansions which were calculated as (cells harvested at t_+7_/cells seeded at t_0_).

#### Tumor target cell lines

Tumor target cell lines, K562‐GFP (ATCC), Nalm6‐GFP (Kite), Nalm6‐CD19 KO‐GFP (Kite) were cultured in RPMI‐1640 + 10% heat‐inactivated FCS at seeding densities of 0.1 × 10^6^ cells mL^−1^ (Nalm6) and 0.3 × 10^6^ cells mL^−1^ (K562) and at 37°C/5% CO_2_.

#### Flow cytometry

Natural killer cells were stained for 20–30 min at room temperature for surface marker expression. Cells were then either analysed directly on a CytoFLEX S (Beckman Coulter, equipped with a 405‐nm, 488‐nm, 561‐nm and 638‐nm laser) or stained for secondary antibodies for an additional 10 min. Cells were then fixed and permeabilized for 20 min using the Foxp3/Transcription Factor Staining Buffer Set (eBioscience). After fixation, cells were intracellularly stained for an additional 30 min before being analysed on a BD Symphony (equipped with a 355‐nm, 405‐nm, 488‐nm, 561‐nm and 637‐nm laser). Data were analysed using FlowJo version 9.9.6 and 10.5.3 (BD Bioscience). The following antibodies were used: CD16 – BUV496, CD56 – BUV563, NKG2A – BB700, CD235a – BV421, CD158e1 (KIR3DL1) – BV711, CD226 (DNAM‐1) – PE, CD56 – FITC, LDLR – PE from BD Bioscience; CD159c (NKG2C) – VioBright FITC, KIR2D – biotin, CD158e/k (KIR3DL1/L2) – biotin, NKp46 – PE‐Vio770 from Miltenyi Biotec; CD158b1/b2 (KIR2DL2/L3/S2) – APC, CD158a,h (KIR2DL1/S1) – PE‐Cy7 from Beckman Coulter; Granzyme B – AF700, CD57 – BV605, CD14 – BV650, CD19 – BV650, CD3 – BV650, KLRG1 – PE, CD2 – Pacific Blue, NKG2D – BV605, CD38 – BV711 from Biolegend; CD19 CAR‐APC from Kite, a Gilead company; Live/Dead Fixable Aqua Dead Cell Stain from Invitrogen; Streptavidin – AF647 from ThermoFisher.

### Functional assays

#### Natural cytotoxicity

NK cells and target cells were seeded at three different effector:target (E:T) cell ratios (1:1, 1:2. 1:4) in RPMI‐1640 + 10% heat‐inactivated FCS + 5 ng mL^−1^ IL‐15 in a black‐walled 96F‐bottom plate. A total of 60 000 cells were seeded in 200 μL of media in duplicates and placed in an IncuCyte S3 (Satorius). K562‐GFP cells were used as target cells. The plate was scanned every 1.5–2 h for 24 h, with the first scan being 30 min after seeding to allow cells to adhere to the plate surface. An object mask was used to identify viable K562‐GFP target cells in the well and the object count per well was normalised to the target cells only condition to plot the percentage of target cells remaining in each well over time.

#### On‐target cytotoxicity

NK cells and target cells were seeded at two different E:T ratios (1:2, 1:4) in RPMI‐1640 + 10% heat‐inactivated FCS + 5 ng mL^−1^ IL‐15 in a black‐walled 96F‐bottom plate. A total of 60 000 cells were seeded in 200 μL of media in duplicates and placed in an IncuCyte S3. Cell number was not adjusted to percentage of transduction for any of the samples. Nalm6‐GFP and Nalm6‐CD19 KO‐GFP cells were used as target cells. The plate was scanned every 2 h for 48 h, with the first scan being 30 min after seeding to allow cells to adhere to the plate surface. An object mask was used to identify viable GFP+ target cells in the well and the object count per well was normalised to the target cells only condition (GFP+) to plot the percentage of target cells remaining in each well over time. For the serial challenge assay, 15 000 NK cells were seeded with 30 000 target cells. 10 000 fresh target cells were added every 24 h for the next three subsequent challenges, with 30 000 fresh target cells being added for the final three challenges, taking into account the growth of NK cells over time.

#### Plasmids

The CAR construct (FMC63‐CD28 (hinge)‐CD28 (TM)‐CD28‐CD3) was provided by Kite Pharma and cloned into the packaging vector pRRL alongside different promoters (EF1a, PGK, CMV, SFFV, MSCV) (Addgene), VSV‐G (Addgene) and BaEV (gift from Els Verhoeyen) were used as envelope proteins for virus production.

#### Viral production and titration

HEK293T cells were transfected using effectene (Qiagen) and 2nd generation viral packaging (psPAX), with Opti‐MEM + 0.1% heat‐inactivated FCS used as viral collection media. Viral supernatant was filtered (0.45 μm) and concentrated using ultracentrifugation (Thermo Scientific Sorvall LYNX 6000, 38 000 *g*, 2 h). Concentrated virus was stored at −80°C. Each viral batch was titrated on CD19 expression on HEK293T cells to determine the viral titre (transduction units mL^−1^ of viral stock). Briefly, HEK293T cells were transduced using a serial dilution of concentrated virus; 72 h post‐transduction, CD19 expression was detected by flow cytometry (CytoFLEX S (Beckman Coulter) and virus titre was calculated as following: Titre (TU mL^−1^) = # cells × %CD19^+^)/volume of virus (mL).

#### Lentiviral transduction

On day 7 of culture, 0.25 × 10^6^ NK cells were seeded in Opti‐MEM + 0.1% heat‐inactivated FCS + 20 ng mL^−1^ IL‐15 + 10 μg mL^−1^ vectofusin (Miltenyi Biotec) in a 48 well plate with or without FMC63‐CD28 (hinge)‐CD28 (TM)‐CD28‐CD3z‐MSCV‐pRRL‐BaEV at a MOI of 5. The plate was centrifuged at 1200 *g* for 1 h at 32°C. After overnight incubation, the cells were harvested, washed and reseeded in GMP‐SCGM + 10% human serum + GlutaMax + 5 ng mL^−1^ IL‐15 in a G‐Rex 48 well plate. For cord blood‐derived NK cells, 250 000 freshly thawed irradiated iKE2‐F9 feeder cells were added, with 330 000 iKE2‐F9 feeder cells being added to PB‐derived NK cells. Four days after transduction, cells were phenotyped to determine transduction efficiency.

#### Cytokine release assay

Supernatant was collected from the on‐target cytotoxicity assay (1:2 E:T, day 14) after 48 h of incubation and frozen in aliquots at −20°C. Twenty human cytokine/chemokines were detected using the Bio‐Plex Pro Human Immunotherapy Panel (20‐plex, Bio‐Rad) with samples detected in duplicates. The plate was analysed on a Biorad Magpix (50plex) and analysed using the BioPlex Data Pro Software (Bio‐Rad).

#### Statistics

Statistical analysis was performed using Prism 8 (GraphPad Software Inc, San Diego). Significance was calculated using either a Mann–Whitney *U*‐test or a Wilcoxon signed rank test, with multiple *t*‐tests (Benjamini) being used when doing multiple two‐group comparisons within the same dataset. Linear regression analysis was performed followed by a Spearman *r* test to determine the significance. *P*‐values: * < 0.05, ** < 0.01, *** < 0.001, **** < 0.0001. Error bars represent standard deviation.

## Author contributions

**Aline Pfefferle:** Conceptualization; formal analysis; investigation; methodology; supervision; visualization; writing – original draft. **Julian Contet:** Formal analysis; investigation; methodology. **Kahlia Wong:** Formal analysis; investigation; methodology. **Charlotte Chen:** Formal analysis; investigation; methodology. **Els Verhoeyen:** Resources. **Chloe K Slichter:** Resources. **Kimberly S Schluns:** Resources. **Joseph Cursons:** Writing – review and editing. **Richard Berry:** Supervision; writing – review and editing. **Iva Nikolic:** Supervision; writing – review and editing. **Jai Rautela:** Conceptualization; funding acquisition; resources; supervision; writing – review and editing. **Nicholas D Huntington:** Conceptualization; funding acquisition; resources; supervision; writing – original draft.

## Conflict of interest

JC, RB and IN report employment with oNKo‐Innate. NDH, JR, IN, JC and RB report stock or other ownership in oNKo‐Innate. NDH serves on an advisory board for Bristol Myers Squibb and Syena. CKS and KSS report employment with Kite, a Gilead company, and stock or other ownership in Gilead Sciences. KSS serves on the advisor board of Obsidian Therapeutics.

## Supporting information


Supplementary figure 1
Supplementary figure 2
Supplementary figure 3
Supplementary figure 4
Supplementary figure 5

## Data Availability

Data are available on request from the authors.
