# Supplementary material for: Optimisation of a primary human CAR‐NK cell manufacturing pipeline
Source: Clin Transl Immunology. 2024 May 2;13(5):e1507. doi: 10.1002/cti2.1507 (PMC11063921; doi:10.1002/cti2.1507)
Supplement: Supplementary file 1 — Supplementary figure 1 Supplementary figure 2 Supplementary figure 3 Supplementary figure 4 Supplementary figure 5 [file CTI2-13-e1507-s001.pdf]

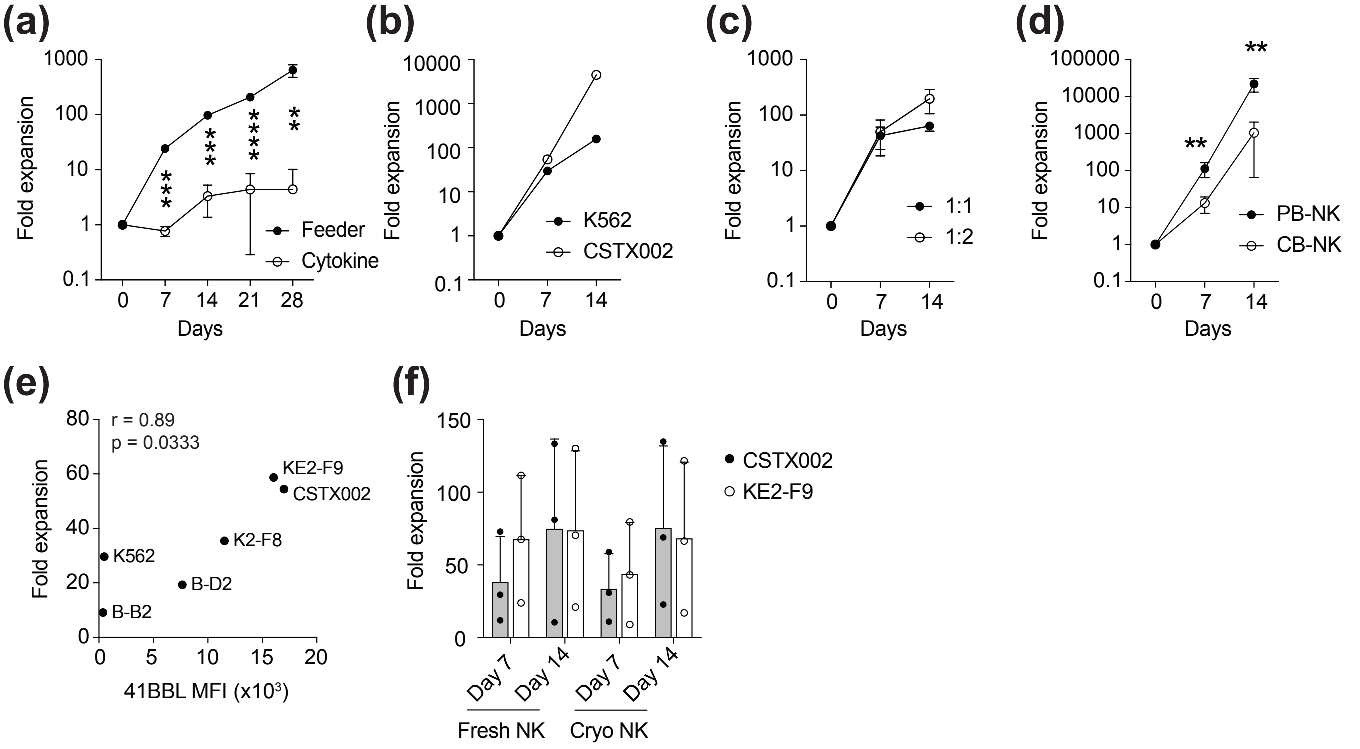

Supplementary figure 1. Optimization of NK cell expansion potential.

Total fold expansion over 28 days of culture comparing PB-derived cytokine-expanded NK cells (20 ng mL^{-1} IL-15) and feeder-expanded NK cells (irradiated K562 + 5 ng mL-1 IL-15 + 25 ng mL^{-1} IL-21) (**a**). Total fold expansion over 14 days of culture comparing PB-derived NK cells expanded using irradiated K562 or CSTX002 as feeder cell line (**b**). Total fold expansion over 14 days of culture comparing PB-drived feeder (CSTX002)-expanded NK cells at varying NK:feeder ratios (**c**). Total fold expansion over 14 days of culture comparing PB- to CB-derived NK cells expanded with CSTX002 feeders at 1:2 ratio (**d**). Relationship between 41BBL expression on different feeder cell clones and the total fold expansion of PB-derived NK cells expanded with these clones after 7 days of culture (**e**). Weekly fold expansion of fresh and cryopreserved PB-derived NK cells expanded with either the CSTX002 or KE2-F9 feeder cell line (**f**). *n = 1-6*.

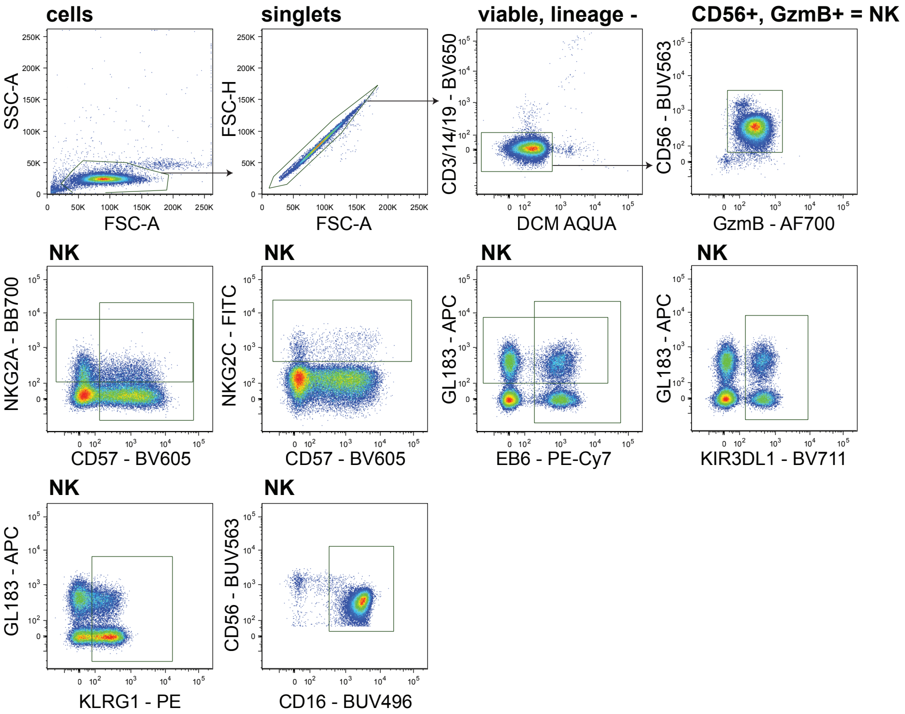

Supplementary figure 2. Phenotyping of NK cells by flow cytometry.
Gating strategy and representative plots of the flow cytometry panel used to characterize NK cell phenotype.

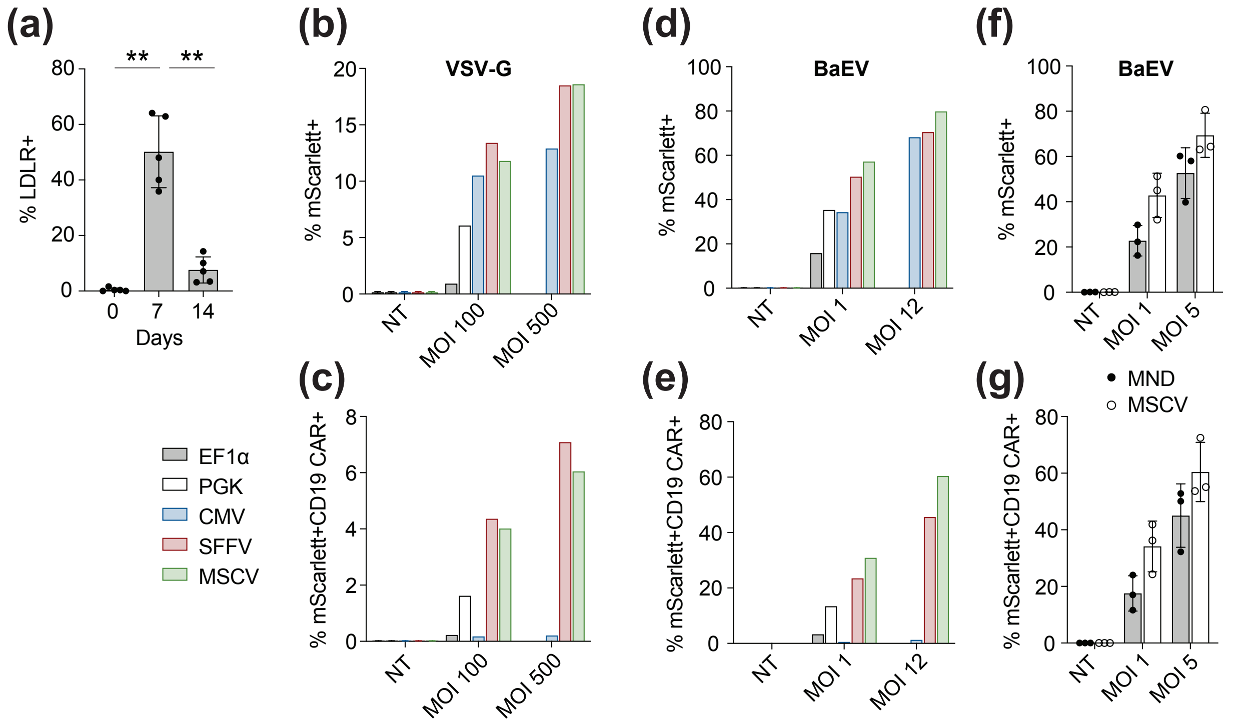

Supplementary figure 3. Optimization of primary NK cell transduction efficiency.
Frequency of LDLR expressing NK cells over 14 days of feeder-expansion (**a**). Frequency of mScarlett+ (**b, d, f**) or CD19 CAR+mScarlett+ (**c, e, g**) NK cells after transduction with VSV-G (**b-c**) or BaEV (**d-g**) pseudotyped virus using different promoters and varying MOIs. *n = 1-5*.

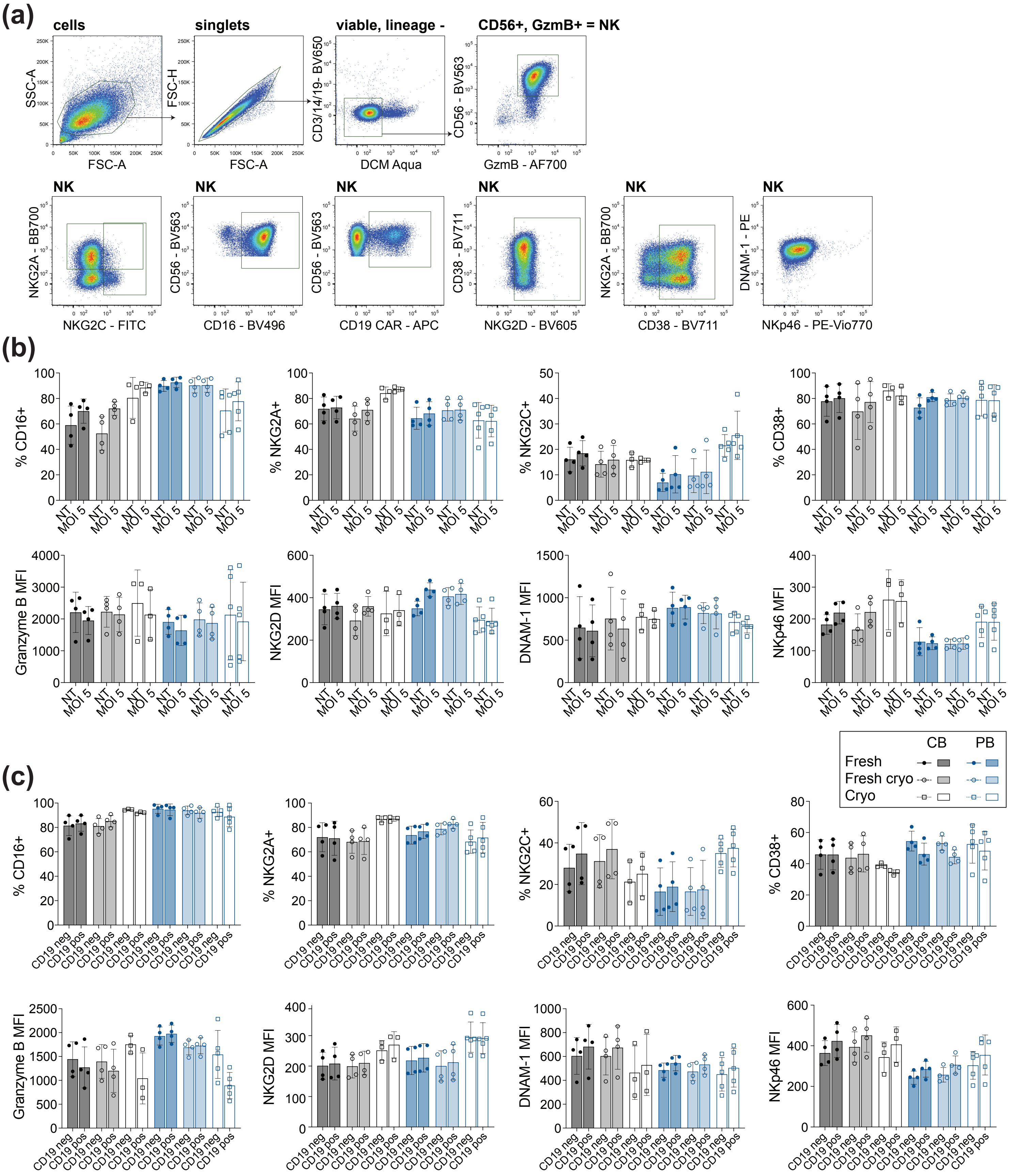

Supplementary figure 4. Phenotyping of CAR-NK cells by flow cytometry.
Gating strategy and representative plots of the flow cytometry panel used to characterize CAR-NK cell phenotype (**a**). Characterization of surface receptor expression and intracellular Granzyme B levels of non-transduced (NT) and transduced (MOI 5) NK cells on day 11 of culture (**b**). Characterization of surface receptor expression and intracellular Granzyme B levels of transduced NK cells on day 21 of culture, stratified based on CAR expression (CAR neg / pos) (**c**). *n = 3-5*.

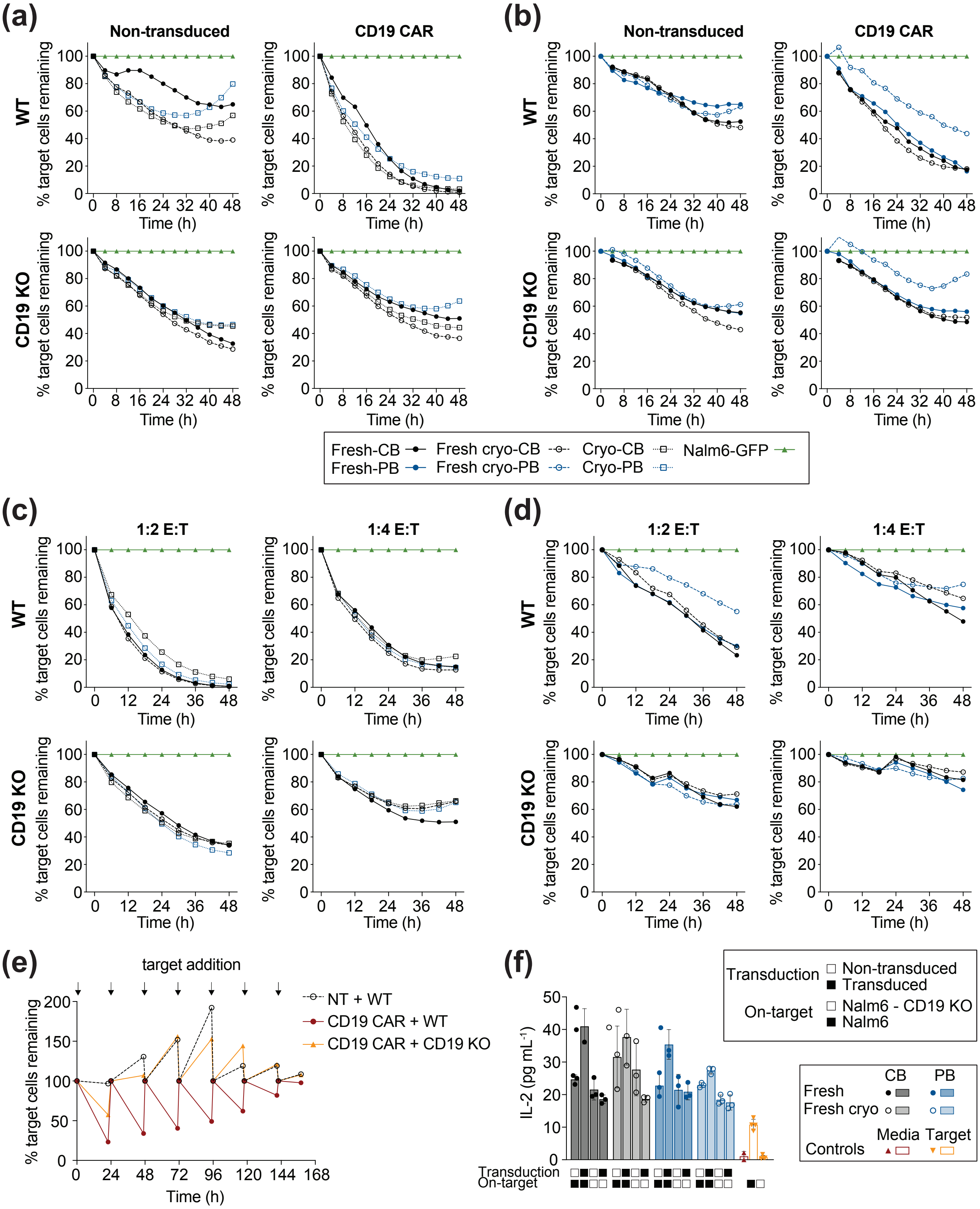

Supplementary figure 5. Functionality of CAR-NK cells from varying sources.
Percentage of target cells (WT or CD19KO Nalm6-GFP), enumerated by object count and normalized to target cells only (Nalm6-GFP), remaining over 48h of co-culture with day 14 non-transduced (NT) or transduced (CD19 CAR) NK cells, with viral batch 1 (**a**) or 2 (**b**), from varying sources at a 1:4 E:T ratio. Percentage of target cells (WT or CD19KO Nalm6-GFP), enumerated by object count and normalized to target cells only (Nalm6-GFP), remaining over 48h of co-culture with 21 day expanded CAR+ NK cells, transduced with viral batch 1 (**c**) or 2 (**d**), at E:T ratios of 1:2 and 1:4. Percentage of targets cells (WT or CD19 KO-Nalm6-GFP), enumerated by object count and normalized to previous timepoint of addition (arrows), remaining over each 24h cytotoxicity window (serial challenge, 1:2 E:T) after a total of 7 challenges (**e**). Quantification of cytokines produced during a 48h cytotoxicity assay by day 14 expanded transduced and non-transduced NK cells against WT and CD19 KO Nalm6 target cells, stratified based on NK cell source (**f**). *n = 2-5*.
